# Impact of cardiometabolic multimorbidity and ethnicity on cardiovascular/renal complications in patients with COVID-19

**DOI:** 10.1136/heartjnl-2021-320047

**Published:** 2021-12-15

**Authors:** Tom Norris, Cameron Razieh, Francesco Zaccardi, Thomas Yates, Nazrul Islam, Clare L Gillies, Yogini V Chudasama, Alex V Rowlands, Melanie J Davies, Gerry P McCann, Amitava Banerjee, Carolyn S P Lam, Annemarie B Docherty, Peter JM Openshaw, J Kenneth Baillie, Malcolm Gracie Semple, Claire Alexandra Lawson, Kamlesh Khunti, ISARIC4C investigators, J Kenneth Baillie

**Affiliations:** 1 Leicester Real World Evidence Unit, Leicester General Hospital, Leicester, UK; 2 Diabetes Research Centre, University of Leicester, Leicester, Leicestershire, UK; 3 Big Data Institute, University of Oxford, Oxford, Oxfordshire, UK; 4 Department of Cardiovascular Sciences, University of Leicester, Leicester, UK; 5 Farr Institute of Health Informatics Research, University College London, London, UK; 6 Department of Cardiology, National Heart Centre Singapore, Singapore; 7 Usher Institute of Population Health Sciences and Informatics, The University of Edinburgh, Edinburgh, Edinburgh, UK; 8 Imperial College London, London, UK; 9 The Roslin Institute, The University of Edinburgh, Easter Bush Campus, Midlothian, UK; 10 University of Liverpool, Liverpool, UK

**Keywords:** COVID-19, epidemiology, risk factors

## Abstract

**Objective:**

Using a large national database of people hospitalised with COVID-19, we investigated the contribution of cardio-metabolic conditions, multi-morbidity and ethnicity on the risk of in-hospital cardiovascular complications and death.

**Methods:**

A multicentre, prospective cohort study in 302 UK healthcare facilities of adults hospitalised with COVID-19 between 6 February 2020 and 16 March 2021. Logistic models were used to explore associations between baseline patient ethnicity, cardiometabolic conditions and multimorbidity (0, 1, 2, >2 conditions), and in-hospital cardiovascular complications (heart failure, arrhythmia, cardiac ischaemia, cardiac arrest, coagulation complications, stroke), renal injury and death.

**Results:**

Of 65 624 patients hospitalised with COVID-19, 44 598 (68.0%) reported at least one cardiometabolic condition on admission. Cardiovascular/renal complications or death occurred in 24 609 (38.0%) patients. Baseline cardiometabolic conditions were independently associated with increased odds of in-hospital complications and this risk increased in the presence of cardiometabolic multimorbidity. For example, compared with having no cardiometabolic conditions, 1, 2 or ≥3 conditions was associated with 1.46 (95% CI 1.39 to 1.54), 2.04 (95% CI 1.93 to 2.15) and 3.10 (95% CI 2.92 to 3.29) times higher odds of any cardiovascular/renal complication, respectively. A similar pattern was observed for all-cause death. Compared with the white group, the South Asian (OR 1.19, 95% CI 1.10 to 1.29) and black (OR 1.53 to 95% CI 1.37 to 1.72) ethnic groups had higher risk of any cardiovascular/renal complication.

**Conclusions:**

In hospitalised patients with COVID-19, cardiovascular complications or death impacts just under half of all patients, with the highest risk in those of South Asian or Black ethnicity and in patients with cardiometabolic multimorbidity.

## Background

As of April 2021, SARS-CoV-2 has infected over 140 million people and claimed over 3 million lives worldwide. After infection, the course of the disease (COVID-19) varies, ranging from asymptomatic mild infection to severe complications and death. People who require hospital admission have the worse outcomes, with a mortality risk of 10%–26% in USA and the UK.^1 2^

Individuals from non-Caucasian ethnic groups or with multiple chronic conditions have been found to be susceptible to severe COVID-19 disease, in-hospital cardiovascular complications and death.^3^ In particular, impaired cardiometabolic health has emerged as an important accelerator of severe COVID-19 disease. Over 25% of patients hospitalised with COVID-19 have cardiometabolic conditions, including hypertension, obesity, diabetes and chronic cardiac disease.^4^ These morbidities have been described as the primary chronic conditions that lead to worsening COVID-19 outcomes.^5 6^ In addition to the baseline risk associated with cardiometabolic conditions, recent studies have shown that COVID-19 can cause acute cardiovascular injury including arrhythmias, cardiac arrest, myocardial infarction and heart failure.^7 8^ These insults can in turn lead to chronic cardiovascular damage or death, even in those without existing cardiovascular disease.^9 10^ Possible mechanisms linking COVID-19 with cardiovascular complications include the release of cytokines (‘cytokine storm’), dysregulation of the renin-angiotensin-aldosterone system and coagulation systems, and plaque rupture during the acute infection phase.^11^

Because people with COVID-19 often have a combination of different high-risk characteristics, it can be difficult to know which are the most important or whether some or all of the higher risk of COVID-19 complications in people who are non-Caucasian is explained by their cardiometabolic conditions. In order to unpick the relative importance of the various factors contributing to adverse COVID-19 prognosis, large, well-phenotyped samples are required, with good coverage of these contributing factors. Accordingly, this study investigated the contribution of multiple cardiometabolic conditions and patient ethnicity, to the risk of cardiovascular/renal complications and death, in a large nationally representative sample of people hospitalised with COVID-19.

## Methods

### Population

We used data from the International Severe Acute Respiratory and emerging Infections Consortium (ISARIC) WHO Clinical Characterisation Protocol UK (CCP-UK) for severe emerging infection. Developed by ISARIC and WHO in 2009, the protocol was reactivated on 17 January 2020 in response to the SARS-CoV-2 pandemic. The protocol and all study materials for this actively recruiting prospective cohort can be accessed online (https://isaric4c.net). The ISARIC WHO CCP-UK study was registered at https://www.isrctn.com/ISRCTN66726260 and designated an Urgent Public Health Research Study by the National Institute for Health Research.

### Sample

Patients aged ≥18 years who were admitted to hospital between 6 February 2020 and 16 March 2021 with a confirmed COVID-19 diagnosis were included. Confirmation of SARS-CoV-2 was done using reverse transcriptase PCR. Highly suspected cases were eligible for inclusion, given that SARS-CoV-2 was an emergent pathogen at the time of protocol activation and laboratory confirmation was dependent on local availability of testing. The enrolment criterion ‘high likelihood of infection’ reflects that a preparedness protocol cannot assume that a diagnostic test will be available for an emergent pathogen. Site training emphasises that only patients who tested positive for COVID-19 were eligible for enrolment. All patients who provided biological samples were required to provide informed, written consent. If patients only provided routinely collected clinical data, written consent was not required (further details can be found in online supplemental text S1).

10.1136/heartjnl-2021-320047.supp1 Supplementary data

Patients who had missing outcome or sex data or were recipients of an organ transplant, immunosuppression therapies or had rare diseases likely to significantly increase the risk of infections (such as severe combined immunodeficiency) were excluded. As of 16 March 2021, 140 975 patients were included in the CCP-UK Study of which 138 455 (98.2%) had a confirmed diagnosis of COVID-19. Of these, 65 624 (46.6%) had the relevant outcome and covariate data for analysis. Online supplemental figure S1 shows how the final sample was derived. Differences in a number of covariates between those included and excluded are provided in online supplemental table S1) and are summarised using standardised differences which are less dependent on the sample size of each group.^12^

**Figure 1:**
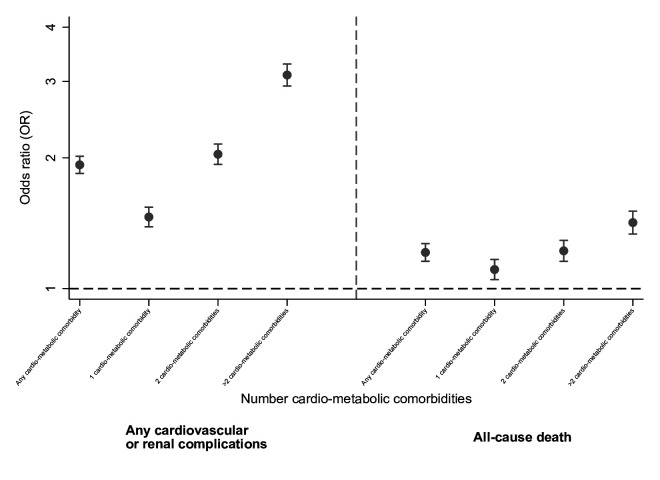
Associations between baseline cardiometabolic multimorbidity and (1) Any in-hospital cardiovascular/renal complication and (2) All-cause death (adjusted for sex, age and ethnicity).

### Exposures

#### Cardiometabolic comorbidities

Data on baseline cardiometabolic comorbidities including hypertension, obesity, diabetes, chronic cardiac conditions and chronic kidney disease were extracted from clinical records. Further information regarding how these variables were classified can be found in online supplemental text S1. We created a dichotomous variable representing the presence of any of the baseline cardiometabolic comorbidities. In addition, to assess the effect of cardiometabolic multimorbidity on outcomes, we created a count variable, summing the number of cardiometabolic morbidities and categorising them as 1, 2 and ≥3.

#### Ethnicity

Ethnicity was coded into four groups, reflecting the most prevalent ethnic groups in the 2011 census in England and Wales,^13^ as follows: white, South Asian, black and other (East and West Asian, Arab, Latin American, Aboriginal/First Nations, other).

#### Covariates

Sex was coded as female/male. Age was measured to the nearest year using the difference between date of birth and the admission date.

### Outcomes

The primary outcomes were (1) A dichotomous variable representing any in-hospital cardiovascular or renal complication; and (2) All-cause death. In-hospital cardiovascular complications were defined clinically in patient healthcare records and extracted by local investigators and included stroke, heart failure, arrhythmia, ischaemia, cardiac arrest and coagulation disorders (abnormal coagulation identified by abnormal prothrombin time or activated partial thromboplastin time). Due to the intrinsic nature of cardiovascular and renal dysfunction we also included ‘acute renal injury’ in the list of complications. These individual cardiovascular/renal complications were considered secondary outcomes. Further information regarding how these variables were defined can be found in online supplemental text S1.

### Statistical analysis

Descriptive statistics are presented as number (%) and median (25th and 75th centiles) for categorical and continuous data, respectively, with differences between those with and without cardiovascular complications in these characteristics examined using standardised differences. To investigate the relationship between cardiometabolic comorbidities, ethnicity and outcomes, we constructed three models.

Model 1: Unadjusted associations between the exposures: (1) ‘*any*’ cardiometabolic comorbidity, (2) Cardiometabolic multimorbidity count and (3) Ethnic group, and the primary outcomes: (1) ‘*any*’ in-hospital cardiovascular/renal complication; (2) In-hospital death and the secondary outcomes: each individual cardiovascular/renal complication.

Model 2: Model 1 exposures (1–2), adjusted for age, sex and ethnicity and Model 1 exposures (3), adjusted for age, sex and baseline cardiometabolic comorbidities. Age was entered into the model as a restricted cubic spline, with four knots placed at the 20th, 40th, 60th and 80th centiles of the age distribution, with the reference value set to the mean age of the sample (70 years of age). This was repeated for the secondary outcomes.

Model 3: To investigate whether the relationship between baseline cardiometabolic comorbidities and *‘any’* in-hospital complications differed according to ethnicity status, we entered first-order interaction terms into Model 2, between ethnic group and ‘*any*’ cardiometabolic comorbidity and between ethnic group and the multimorbidity count variable. Interactions were formally assessed using the Wald test, with p<0.05 indicating a significant interaction term. Predicted probabilities for each interaction term and outcome were estimated and plotted using Stata’s postestimation ‘margins’ and ‘marginsplot’ commands.

### Supplementary analyses

We repeated Models 1 and 2 replacing the dichotomous ‘*any*’ cardio-metabolic comorbidity exposure variable with each individual baseline cardio-metabolic comorbidity. Furthermore, we repeated Models 1 to 3 using a composite outcome representing ‘any complication or all-cause death’ to account for the competing risk of death in assessing in-hospital complications.

A final set of models was constructed to investigate the separate and combined associations between baseline cardiometabolic comorbidities, in-hospital cardiovascular/renal complications and death. For this model, we created a single variable which categorised patients into four groups: patients with no baseline cardiometabolic morbidities or in-hospital cardiovascular/renal complications (reference group); patients with baseline cardiometabolic morbidities only; patients with in-hospital cardiovascular/renal complications only, and patients with both baseline cardiometabolic morbidities and in-hospital cardiovascular/renal complications. Logistic regression was then used to estimate the association between this categorical variable and death, with adjustment for sex, age and ethnicity.

All analyses were conducted in Stata V.16 (Stata Corp, College Station, Texas, USA). Data are reported with 95% CI unless reported otherwise.

### Patient and public involvement

This was an urgent public health research study in response to a public health emergency of international concern. Patients or the public were not involved in the design, conduct or reporting of this rapid response research.

## Results

The sample comprised 34 891 men (53.2%), had a median age of 73 years (IQR: 58–84) and 55 588 (84.7%) of individuals were of ‘White’ ethnicity (table 1). Over two-thirds of individuals (44 598; 68.0%) reported at least one cardiometabolic condition on admission, with 30.5%, 22.8% and 14.7% of the sample reporting one, two or more than two baseline cardiometabolic conditions, respectively; 3612 (5.5%) individuals required an invasive ventilation procedure (tracheal intubation or tracheostomy) while in hospital. More than a quarter of the sample experienced a cardiovascular/renal complication while in hospital (n=16 628, 25.3%), of which 9702 (58.3%) patients survived and 6926 (n=41.7%) died. Of patients who experienced a cardiovascular/renal complication 2133 (12.8%) required an invasive ventilation procedure while in hospital, compared with 1479 (3.0%) of those who did not. Of the patients, 8293 (16.9%) died who did not experience a cardiovascular/renal complication or in-hospital death, resulting in a total of 15 219 (23.2%) patient deaths. Of the patients, 1685 (46.7%) who had required an invasive ventilation procedure while in hospital died, compared with 13 478 (21.8%) of those who did not require such intervention.

**Table 1:** Patient characteristics, stratified by presence of cardiovascular complications (n=65 624)

	Patients not experiencing cardiovascular complication (n=48 996)	Patients experiencing cardiovascular complication (n=16 628)	Standardised differences^*^
*Sex*			
Male	25 219 (51.5)	9 672 (58.2)	0.13
Female	23 777 (48.5)	6 956 (41.8)	
Age on admission (years)	71 (55, 83)	78 (66, 86)	0.40
*Ethnicity*			
White	41 234 (84.2)	14 354 (86.3)	0.07
South Asian	3 056 (6.2)	868 (5.2)	
Black	1 248 (2.6)	461 (2.8)	
Other	3 458 (7.1)	945 (5.7)	
*In-hospital cardiovascular complications*			
Arrhythmia	–	4 446 (27.4)	
Cardiac ischaemia	–	857 (5.3)	
Cardiac arrest	–	1 370 (8.4)	
Coagulation complications	–	2 088 (12.9)	
Stroke	–	1 043 (6.4)	
Heart failure	–	2 297 (14.1)	
Renal injury	–	9 968 (60.5)	
*Baseline cardiometabolic comorbidities*			
Diabetes	8 276 (16.9)	4 152 (25.0)	0.20
Chronic cardiac disease	12 872 (26.3)	7 265 (43.7)	0.37
Hypertension	20 552 (42.0)	9 425 (56.7)	0.30
Chronic kidney disease	6 126 (12.5)	4 362 (26.2)	0.35
Obesity	6 030 (12.3)	2 702 (16.3)	0.11

Data are reported as n(%) for categorical variables and median (IQR) for continuous variables.

*Standardised difference = difference in means or proportions divided by SE; imbalance defined as absolute value >0.10.

In total, 24 921 (38.0%) of the sample experienced either a cardiovascular/renal complication or died.

### Primary outcomes: any cardiovascular/renal complications and all-cause death

#### Baseline cardiometabolic comorbidity

Unadjusted associations between baseline cardiometabolic comorbidity, multimorbidity, cardiovascular/renal complications and all-cause death are shown in online supplemental table S2. Having any baseline cardiometabolic comorbidity was associated with more than twice the odds of experiencing a cardiovascular/renal complication (OR 2.54, 95% CI 2.43 to 2.65). The association between baseline cardio-metabolic comorbidities and increased risk of in-hospital cardiovascular/renal complications remained following adjustment for age, sex and ethnicity (table 2, figure 1). For example having any baseline cardio-metabolic comorbidity was associated with 1.93 (95% CI 1.84 to 2.02) times higher odds of experiencing a cardiovascular/renal complication. In terms of multimorbidity, individuals with one, two or more than two baseline comorbidities had 1.46 (95% CI 1.39 to 1.54), 2.04 (95% CI 1.93 to 2.15) and 3.10 (95% CI 2.92 to 3.29) times higher odds, respectively, of experiencing a cardiovascular/renal complication.

**Table 2:** Association between baseline cardiometabolic comorbidities, cardiovascular/renal complications and all-cause death

	Primary outcomes		Secondary outcomes						
	Any complication	All-cause death	Heart failure	Arrhythmia	Cardiac ischaemia	Cardiac arrest	Coagulation complications	Stroke	Renal injury
	OR (95% CI)		OR (95% CI)	OR (95% CI)	OR (95% CI)	OR (95% CI)	OR (95% CI)	Or (95% CI)	Or (95% CI)
Any cardiometabolic comorbidity (ref: No)^*^	1.93 (1.84 to 2.02)	1.21 (1.16 to 1.27)	4.19 (3.56 to 4.94)	1.69 (1.56 to 1.83)	2.61 (2.11 to 3.23)	1.24 (1.09 to 1.42)	1.49 (1.34 to 1.66)	1.19 (1.02 to 1.38)	2.08 (1.96 to 2.20)
Number of cardiometabolic comorbidities (ref: 0)^*^									
1	1.46 (1.39 to 1.54)	1.11 (1.05 to 1.17)	2.23 (1.86 to 2.67)	1.44 (1.31 to 1.57)	1.88 (1.48 to 2.39)	1.04 (0.89 to 1.22)	1.37 (1.21 to 1.54)	1.19 (1.00 to 1.41)	1.52 (1.42 to 1.62)
2	2.04 (1.93 to 2.15)	1.22 (1.16 to 1.29)	4.59 (3.85 to 5.46)	1.74 (1.58 to 1.91)	2.60 (2.05 to 3.30)	1.40 (1.19 to 1.63)	1.38 (1.21 to 1.58)	1.17 (0.98 to 1.41)	2.18 (2.04 to 2.33)
>2	3.10 (2.92 to 3.29)	1.42 (1.34 to 1.51)	8.01 (6.73 to 9.53)	2.23 (2.02 to 2.47)	4.24 (3.35 to 5.38)	1.46 (1.23 to 1.73)	2.03 (1.77 to 2.32)	1.21 (0.99 to 1.48)	3.41 (3.18 to 3.66)

*Separate models, adjusted for sex, age and ethnicity.

Adjusted associations between baseline cardiometabolic comorbidities, multimorbidity and all-cause death are shown in table 2. The presence of any baseline cardiometabolic comorbidity was associated with 1.21 (95% CI 1.16 to 1.27) times higher odds of death. Progressively higher odds of death were observed with an increasing number of baseline cardiometabolic comorbidities, such that individuals with one, two or more than two comorbidities demonstrated odds which were 1.11 (95% CI 1.05 to 1.17), 1.22 (95% CI 1.16 to 1.29) and 1.42 (95% CI 1.34 to 1.51) times higher than individuals without baseline comorbidities.

#### Ethnicity

Unadjusted associations between ethnic group and cardiovascular/renal complications are shown in online supplemental table S2. Following adjustment for age, sex and baseline cardiometabolic comorbidities, patients of South Asian (OR 1.13, 95% CI 1.04 to, 1.22) or black (1.46, 95% CI 1.30 to 1.63) ethnicity demonstrated increased odds of experiencing a cardiovascular/renal complication, compared with the white ethnic group (table 3). However, we did not find any strong evidence in support of an interaction between ethnicity and cardiometabolic comorbidities (online supplemental tables S3 and S4), (online supplemental figures S2 and S3).

**Table 3:** Association between ethnicity, cardiovascular/renal complications and all-cause death

	Primary outcome		Secondary outcome						
	Any complication	All-cause death	Heart failure	Arrhythmia	Cardiac ischaemia	Cardiac arrest	Coagulation complications	Stroke	Renal injury
	OR (95% CI)		OR (95% CI)	OR (95% CI)	OR (95% CI)	OR (95% CI)	OR (95% CI)	OR (95% CI)	OR (95% CI)
*Ethnicity (ref: white*)									
*South Asian*	1.13 (1.04 to 1.22)	1.27 (1.16 to 1.40)	0.97 (0.78 to 1.22)	0.82 (0.70 to 0.95)	1.49 (1.13 to 1.98)	1.83 (1.49 to 2.23)	0.97 (0.81 to 1.17)	1.10 (0.82 to 1.47)	1.19 (1.07 to 1.31)
*Black*	1.46 (1.30 to 1.63)	1.18 (1.03 to, 1.36)	1.02 (0.75 to 1.41)	0.83 (0.67 to 1.04)	0.78 (0.45 to 1.36)	2.12 (1.62 to 2.79)	1.32 (1.04 to 1.68)	0.96 (0.61 to 1.51)	1.77 (1.56 to 2.02)
*Other*	1.05 (0.97 to 1.14)	1.07 (0.98 to 1.16)	1.00 (0.82 to 1.23)	0.86 (0.74 to 0.99)	1.40 (1.07 to 1.83)	1.17 (0.94 to 1.47)	0.96 (0.81 to 1.15)	1.15 (0.88 to 1.49)	1.10 (1.00 to 1.21)

Adjusted for sex, age and baseline cardiometabolic comorbidities.

After adjusting for sex, age and baseline cardiometabolic comorbidities, the South Asian (OR 1.27; 95% CI 1.16 to 1.40) and black ethnic groups (OR 1.18; 95% CI 1.03 to 1.36) were at significantly higher risk of all-cause death compared with the white group (table 3). The magnitude of these associations remained largely unchanged following adjustment for baseline cardiometabolic comorbidities.

### Secondary outcomes: individual cardiovascular/renal complications

#### Baseline cardio-metabolic comorbidity

**Figure 2:**
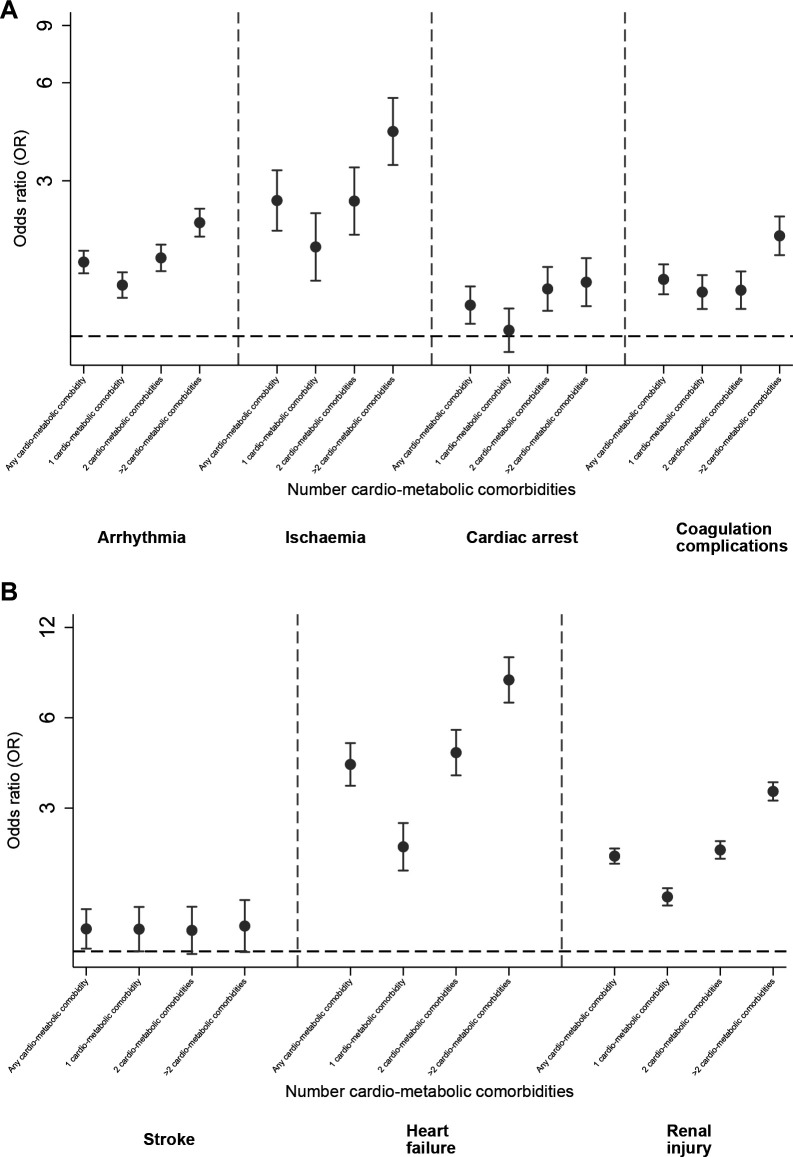
(A) Associations between baseline cardiometabolic multimorbidity and each in-hospital cardiovascular/renal complication (adjusted for sex, age and ethnicity). (B) Associations between baseline cardiometabolic multimorbidity and each in-hospital cardiovascular/renal complication (adjusted for sex, age and ethnicity).

The association between baseline comorbidities and individual complications can been seen in table 2 and figure 2A,B. A pattern of increasing odds of complications with an increasing number of baseline cardio-metabolic conditions was observed for heart failure, arrhythmia, cardiac ischaemia and acute renal injury.

#### Ethnicity

The South Asian group was almost twice as likely to experience cardiac arrest (OR 1.83, 95% CI 1.49 to 2.23) and almost 50% more likely to experience cardiac ischaemia (OR 1.49, 95% CI 1.13 to 1.98) than the white group. The black group was more than twice as likely to experience cardiac arrest (OR 2.12, 95% CI 1.62 to 2.79) and over 75% more likely to experience acute renal injury (OR 1.77, 95% CI 1.56 to 2.02), compared with the white group (table 3).

### Supplementary analyses

In adjusted models, chronic kidney disease exhibited the strongest association with the likelihood of experiencing any cardiovascular/renal complication (OR 2.02, 95% CI 1.93 to 2.11), while obesity conferred the greatest odds of death (OR 1.34, 95% CI 1.26 to 1.43) (online supplemental table S5).

We observed a consistent pattern of associations between cardiometabolic comorbidity, ethnicity and the composite outcome representing ‘cardiovascular/renal complication or all-cause death’ (online supplemental tables S6 and S7 and online supplemental figures S4 and S5).

Compared with patients with no baseline cardiometabolic conditions or in-hospital complications, patients with baseline cardiometabolic comorbidities only had 20% higher odds of death (OR 1.20, 95% CI 1.13 to 1.27). Those with a baseline cardiometabolic condition and experiencing an in-hospital complication had over three times higher odds of death, compared with those with neither (OR: 3.17, 95% CI 2.98 to 3.37). However, those who had no baseline cardiometabolic conditions, but who subsequently experienced an in-hospital cardiovascular/renal complication, had almost four times higher odds of death, relative to patients with neither (OR 3.92, 95% CI 3.57 to 4.29).

## Discussion

In 65 624 hospitalised patients with COVID-19, 68% had at least one cardiometabolic comorbidity on admission and 38% experienced an in-hospital cardiovascular/renal complication or died. The presence of cardiometabolic conditions and multimorbidity on admission increased the risk of experiencing in-hospital cardiovascular/renal complications and death. Risk of in-hospital complications were also increased in people from South Asian and black ethnicity groups, independent of their cardiometabolic status on admission to hospital.

Several studies have previously reported that the presence of chronic conditions is associated with a more severe course and progression of COVID-19, ultimately culminating in an increased risk of mortality.^14–16^ There has been less attention on the short-term morbidity events which are likely associated with significant long-term morbidity. The most common complication observed was acute renal injury, which is associated with increased long-term risk of mortality, requirement for dialysis and an increase in cardiovascular events.^17–19^ While cardiac ischaemia was the least common cardiovascular/renal complication observed (5.3%), if left untreated, the ischaemia can progress to ischaemic heart disease which is the leading cause of death globally.^20^

Our study extends the findings from previous studies,^14–16 21 22^ which were largely based on either significantly smaller samples or in single healthcare settings or cities, and which demonstrated an increased risk of severe COVID-19 illness in those with pre-existing conditions. While associations between individual comorbidities and in-hospital complications differed in their magnitude, a consistent finding was the increasing risk of all complications, aligned with the increasing number of cardio-metabolic morbidities at baseline. This multimorbidity association was strongest for the risk of heart failure, with those reporting more than two cardio-metabolic conditions having more than eight times higher odds, compared with patients with no cardio-metabolic conditions. Previous studies have suggested that acute heart failure can be the primary presenting manifestation of COVID-19 infection.^23^ An early study in China observed that 40% of fatalities in hospitalised critically ill patients with COVID-19 were associated with myocardial damage and heart failure.^24^ Whether this heart failure represents a new cardiomyopathy or an exacerbation of previously undiagnosed heart failure is however, unknown.^25^

While we did not observe strong evidence of a modifying effect of ethnicity on the relationship between cardiometabolic comorbidities and in-hospital complications (only), we did find some evidence that the association between cardiometabolic comorbidities and the composite outcome of cardiovascular/renal complications or death, was modified by ethnicity (online supplemental figures S4 and S5). This could be due to a modifying effect of ethnicity on the relationship between cardiometabolic comorbidities and death, which we did observe weak evidence for (p_(interaction)_=0.03, models not shown). Belonging to an ethnic minority group is associated with elevated risk for severe illness from COVID-19,^26 27^ likely resulting from a combination of socioeconomic, cultural/lifestyle factors, genetic predisposition, or pathophysiological differences in susceptibility or response to infection.^27^ Such susceptibilities include a higher prevalence of cardiometabolic conditions including insulin resistance, obesity and hypertension,^28^ though adjustment for baseline comorbidities (eg, diabetes status) did not attenuate the increased risk in these groups.

### Strengths and limitations

These data were collected prospectively across 302 hospitals and represent a large proportion (approximately 40%) of people hospitalised with COVID-19 in the UK, thus increasing the generalisability of our findings. Unlike our study, other smaller, or single-centre studies have typically focused either exclusively on patients who received critical care, or on one type of complication and lack systematic approaches to data collection.^29^ In terms of limitations, the complications and comorbidities that were captured were predefined by a pragmatic outbreak preparedness study protocol which was developed prior to the emergence of COVID-19 and which may lack the granular details which could be instead collected in ad hoc cohort studies or trials (ie, brain natriuretic peptide or echocardiography). All medical conditions, which were clinically defined following the diagnostic procedures deemed relevant by the healthcare professional, were extracted from clinical records and inputted from the local health professionals in charge of each individual’s care. Furthermore, the combined definition of ‘chronic cardiac disease’ combines a variety of conditions (eg, coronary artery disease, heart failure, congenital heart disease, cardiomyopathy and rheumatic heart disease) which may potentially confer distinct and diverging pathophysiological responses to COVID-19. In addition, we did not have data regarding the severity or duration of the cardiometabolic comorbidities experienced, which would have provided a more precise estimation of the burden of cardiometabolic disease at baseline. Relatedly, a lack of sociodemographic and lifestyle factors (eg, smoking habits and physical activity) included in the study protocol means that the possibility of residual confounding biasing our estimates cannot be excluded.

Our study highlights the importance of adequate risk factor control (ie, regular monitoring) in reducing the risk of hospital admission and complications if infected with COVID-19. This is especially important in individuals already at an increased risk of developing cardiometabolic conditions such as those with prediabetes, prehypertension or those who are overweight. Hospitalised patients with COVID-19 who survive after cardiovascular/renal complications are likely to experience long-term morbidity. As such, governments, policy makers, healthcare planners and front-line healthcare workers should anticipate an increased burden placed on health and social care resources, which will be critical to support those who survive COVID-19.

**Key messages**

**What is already known about this subject?**

- Findings from several small and regional studies suggest that certain patient groups admitted to hospital with COVID-19 are more susceptible to in-hospital cardiovascular complications and death.

**What does this study add?**

- In this cohort of 65 624 patients with COVID-19, in-hospital cardiovascular/renal complications or death occurred in 38%. A greater number of cardiometabolic comorbidities on admission and being of black or South Asian ethnicity increased odds of cardiovascular/renal complications. A similar pattern was observed for all-cause death.

**How might this impact on clinical practice?**

- Our findings indicate the prognostic relevance of several risk factors on the risk of in-hospital complications if infected with COVID-19. This is especially important in individuals already at an increased risk of developing cardiometabolic conditions such as those with prediabetes, prehypertension or those who are overweight.

## Data Availability

Data may be obtained from a third party and are not publicly available. Data and analysis scripts are available on request to the Independent Data Management and Access Committee at https://isaric4c.net/sample_access.
