# Supplementary material for: Impact of cardiometabolic multimorbidity and ethnicity on cardiovascular/renal complications in patients with COVID-19
Source: Heart. 2021 Dec 15:heartjnl-2021-320047. doi: 10.1136/heartjnl-2021-320047 (PMC8678560; doi:10.1136/heartjnl-2021-320047)

Supplementary material:

Impact of cardio-metabolic multimorbidity and ethnicity on cardiovascular and renal complications: an observational study in patients hospitalised with COVID-19

Tom Norris, PhD[1,3] tdn9@leicester.ac.uk

Cameron Razieh, MSc[1, 2] cr288@leicester.ac.uk

Francesco Zaccardi, PhD[1, 3] fz43@leicester.ac.uk

Thomas Yates, PhD[1, 2] ty20@leicester.ac.uk

Nazrul Islam, PhD[4, 5] nazrul.Islam@ndph.ox.ac.uk

Clare L Gillies, PhD[1, 3] clg13@leicester.ac.uk

Yogini V Chudasama, PhD[3] yc244@leicester.ac.uk

Alex Rowlands, PhD[1, 2] alex.rowlands@leicester.ac.uk

Melanie J Davies, MD[1, 2] melanie.davies@uhl-tr.nhs.uk

Gerry P McCann, MD[2,4] gpm12@leicester.ac.uk

Ami Banerjee, MD PhD[6] ami.banerjee@ucl.ac.uk

Carolyn Lam, MD PhD[7,8] carolyn.lam@duke-nus.edu.sg

Annemarie B Docherty PhD [9, 10] annemarie.docherty@ed.ac.uk

Peter JM Openshaw, PhD[11] p.openshaw@imperial.ac.uk

J Kenneth Baillie, PhD[12] j.k.baillie@ed.ac.uk

Malcolm G Semple, PhD[13, 14] M.G.Semple@liverpool.ac.uk

ISARIC4C investigators*

Claire A. Lawson*, PhD[3,4] cl417@leicester.ac.uk

Kamlesh Khunti, PhD[1, 3, 15] kk22@leicester.ac.uk

*Joint senior author

Norris T, *et al. Heart* 2021;0:1–9. doi: 10.1136/heartjnl-2021-320047

Contents

Supplementary Text

Text S1. Testing 3

Text S2. Variable definitions 3

Table S1. Characteristics of those included in the final sample (n=65 624) vs those excluded for having incomplete exposure, covariate or outcome data (n= 49 457) 5

Table S2. Univariate associations between presence of any baseline cardio-metabolic morbidities, number of baseline cardio-metabolic multi-morbidities, each individual cardio-metabolic morbidity, ethnicity and in-hospital cardiovascular and renal complications 6

Table S3. Association between any cardio-metabolic morbidity and any cardiovascular or renal complication: effect modification by ethnicity 7

Table S4. Association between cardio-metabolic multi-morbidity and any cardiovascular or renal complication: effect modification by ethnicity 8

Table S5. Association between individual cardio-metabolic morbidities, in-hospital cardiovascular and renal complications and death 9

Table S6. Association between baseline cardio-metabolic comorbidities and 'cardiovascular and renal complications or all-cause death' 10

Table S7. Association between ethnicity and 'cardiovascular and renal complications or all-cause death' 11

Table S8. Associations between baseline cardio-metabolic multi-morbidity, in-hospital cardiovascular and renal complications and death 12

Figure S1. Flow diagram outlining how final sample was derived 13

Figure S2. Association between any cardio-metabolic comorbidity and any cardiovascular or renal complication: modification by ethnicity 14

Figure S3. Association between cardio-metabolic multi-morbidity and any cardiovascular or renal complication: modification by ethnicity 15

Figure S4. Association between any cardio-metabolic comorbidity and 'cardiovascular and renal complications or all-cause death': modification by ethnicity 16

Figure S5. Association between cardio-metabolic multi-morbidity and 'cardiovascular and renal complications or all-cause death': modification by ethnicity 17

Text S1. Testing

The criteria to test for SARS-CoV-2 by PCR were not defined by the study; the decision to test was at the discretion of the clinician attending the patient. To support this, national guidance was provided by PHE and other UK public health agencies that advises who to test based on clinical case definitions for possible COVID-19, and also prioritisation of testing when demand exceeds capacity. In reality, anyone who was admitted to hospital and met the case definition for possible COVID-19 would have been tested, as capacity was never so limiting that hospital patients had to be triaged for testing.

We also included patients who had been admitted for a separate condition but had tested positive for covid-19 during their hospital stay.

Text S2. Variable definitions

Data were directly transcribed from routine healthcare into case report forms hosted on a REDCap database (Research Electronic Data Capture, https://projectredcap.org). Data collection was undertaken by research nurses, administrators and medical students. Detailed demographic and clinical data were collected on admission, with follow-up data on clinical care collected at day 3, 6, and 9 and discharge or status at 28 days if not discharged. The outcome of hospital admission was coded on discharge or death

Baseline cardio-metabolic comorbidities:

Chronic cardiac disease included any of the following: coronary artery disease, heart failure, congenital heart disease, cardiomyopathy and rheumatic heart disease. Obesity was defined by clinical staff, ideally but not necessarily, with an objective measurement of obesity, such as calculation of the body mass index (BMI of 30 or more) or measurement of abdominal girth. Chronic kidney disease was defined based on the presence of any of the following: clinician-diagnosed chronic kidney disease, chronic estimated glomerular filtration rate <60 mL/min/1.73m^2, history of kidney transplantation. Diabetes was defined as pre-existing clinician-diagnosed Type 1 or Type 2 diabetes. Similarly, hypertension was based on a pre-existing clinician diagnosis of hypertension.

Cardiovascular and renal complications:

Norris T, *et al. Heart* 2021;0:1–9. doi: 10.1136/heartjnl-2021-320047

Stroke was based on a clinical diagnosis, with or without supportive radiological findings. Heart failure was defined as failure of the heart to pump a sufficient amount of blood to meet the needs of the body tissues, resulting in tissue congestion and oedema. Cardiac arrhythmia was defined as presence of arrhythmia in those without a previous record of it, confirmed by electrocardiographic monitoring. Cardiac ischaemia was defined as diminished blood and oxygen supply to the heart muscle, also known as myocardial ischemia and was confirmed by an electrocardiogram (showing ischaemic changes, e.g. ST depression or elevation) and/or cardiac enzyme elevation. Cardiac arrest referred to the sudden cessation of cardiac activity with no normal breathing and no signs of circulation. Coagulation disorder was defined as abnormal coagulation identified by abnormal prothrombin time or activated partial thromboplastin time. Acute renal injury was defined as urine volume <0.5 mL/kg/hour for 6 hours or as a creatinine rise which corresponded to the Kidney Disease Improving Global Outcomes (KDIGO) stage I or above definition (increase in serum creatinine by ≥0.3 mg/dL (≥26.5 μmol/L) within 48 hours; increase in serum creatinine to ≥1.5 times baseline, which is known or presumed to have occurred within the prior 7 days)[1].

1. Kellum JA, Lameire N, Aspelin P, Barsoum RS, Burdmann EA, Goldstein SL, Herzog CA, Joannidis M, Kribben A, Levey AS. Kidney disease: improving global outcomes (KDIGO) acute kidney injury work group. KDIGO clinical practice guideline for acute kidney injury. Kidney international supplements 2012;2(1):1-138.

Table S1. Characteristics of those included in the final sample (n=65 624) vs those excluded for having incomplete exposure, covariate or outcome data (n= 49 457)

	**Included in final sample (n=65 624)**		**Excluded due to incomplete data (n=49 457)**		***Standardised differences*** †
	total n		*total n*		
Sex (male, n (%))	65 624	27 725 (56.1)	49 457	34 891 (53.2)	0.06
Age (years, mean (SD))	65 624	69.4 (18.0)	49 457	71.4 (16.3)	0.12
Ethnicity (n (%))					
White British		55 588 (84.7)		41 548 (84.0)	
South Asian	65 624	3 924 (6.0)	49 457	2 759 (5.6)	0.04
Black		1 709 (2.6)		1 488 (3.0)	
Other		4 403 (6.7)		3 662 (7.4)	
Any cardio-metabolic comorbidities on admission (yes, n (%))	65 624	44 598 (68.0)	30 651	29 797 (97.2)	0.84
In-hospital cardiovascular complications (yes, n(%))	65 624	16 628 (25.3)	38 183	10 854 (28.4)	0.07

† Standardized difference = difference in means or proportions divided by standard error; imbalance defined as absolute value greater than 0.10

Table S2. Univariate associations between presence of any baseline cardio-metabolic morbidities, number of baseline cardio-metabolic multi-morbidities, each individual cardio-metabolic morbidity, ethnicity and in-hospital cardiovascular and renal complications

	**Primary outcome**	**Secondary outcome**							
	**Any complication**	**Death**	**Heart failure**	**Arrhythmia**	**Cardiac Ischaemia**	**Cardiac arrest**	**Coagulation complications**	**Stroke**	**Renal injury**
	**OR (95% CI)**	**OR (95% CI)**	**OR (95% CI)**	**OR (95% CI)**	**OR (95% CI)**	**OR (95% CI)**	**OR (95% CI)**	**OR (95% CI)**	**OR (95% CI)**
Any cardio-metabolic comorbidity (ref: No)	2.54 (2.43, 2.65)	2.12 (2.03, 2.22)	6.50 (5.53, 7.63)	2.05 (1.90, 2.22)	3.59 (2.91, 4.42)	1.56 (1.37, 1.77)	1.41 (1.27, 1.55)	1.61 (1.39, 1.86)	2.72 (2.57, 2.87)
Number cardio-metabolic comorbidities (ref: 0)									
1	1.79 (1.70, 1.88)	1.73 (1.65, 1.82)	3.10 (2.59, 3.72)	1.66 (1.52, 1.82)	2.38 (1.88, 3.01)	1.23 (1.06, 1.43)	1.30 (1.15, 1.46)	1.51 (1.28, 1.79)	1.85 (1.73, 1.97)
2	2.72 (2.59, 2.87)	2.30 (2.18, 2.42)	7.26 (6.12, 8.61)	2.13 (1.95, 2.33)	3.62 (2.87, 4.56)	1.77 (1.52, 2.05)	1.26 (1.11, 1.43)	1.65 (1.38, 1.96)	2.87 (2.69, 3.06)
>2	4.23 (4.00, 4.48)	2.77 (2.61, 2.93)	12.84 (10.83, 15.21)	2.78 (2.53, 3.05)	6.09 (4.84, 7.66)	1.91 (1.63, 2.25)	1.86 (1.63, 2.11)	1.75 (1.44, 2.12)	4.61 (4.31, 4.93)
Ethnicity (ref: White)									
South Asian	0.82 (0.75, 0.88)	0.66 (0.60, 0.71)	0.59 (0.47, 0.73)	0.67 (0.58, 0.77)	1.07 (0.81, 1.41)	1.45 (1.19, 1.76)	1.05 (0.87, 1.26)	0.79 (0.60, 1.05)	0.87 (0.79, 0.96)
Black	1.06 (0.95, 1.18)	0.64 (0.56, 0.72)	0.64 (0.47, 0.87)	0.69 (0.55, 0.86)	0.58 (0.33, 1.00)	1.72 (1.31, 2.25)	1.42 (1.12, 1.79)	0.71 (0.46, 1.11)	1.30 (1.15, 1.47)
Other	0.79 (0.73, 0.85)	0.63 (0.58, 0.68)	0.63 (0.51, 0.77)	0.71 (0.62, 0.81)	1.02 (0.78 - 1.33)	0.96 (0.77, 1.20)	1.01 (0.85, 1.21)	0.87 (0.68, 1.13)	0.83 (0.76, 0.91)

Table S3. Association between any cardio-metabolic morbidity and any cardiovascular or renal complication: effect modification by ethnicity

	Any complication
	**OR (95% CI)**
**Ethnicity** *(ref: White)*	1.00
South Asian	1.03 (0.87 , 1.23)
Black	1.27 (0.99 , 1.61)
Other	1.07 (0.92, 1.24)
**Any baseline cardio-metabolic comorbidity** *(ref: No)*	
Yes	1.91 (1.82, 2.01)
**Interaction**	
White # No cardio-metabolic comorbidity *(ref)*	1.00
South Asian # Any cardio-metabolic comorbidity	1.11 (0.92 , 1.36)
Black # Any cardio-metabolic comorbidity	1.20 (0.91 , 1.58)
Other # Any cardio-metabolic comorbidity	0.97 (0.82 , 1.16)
p (interaction)	0.399

*adjusted for age and sex

Table S4. Association between cardio-metabolic multi-morbidity and any cardiovascular or renal complication: effect modification by ethnicity

	**Any complication**
	**OR (95% CI)**
**Ethnicity** *(ref: White)*	1.00
South Asian	1.00 (0.84, 1.19)
Black	1.23 (0.96, 1.56)
Other	1.04 (0.90, 1.21)
**Baseline cardio-metabolic comorbidity**	
0 *(ref)*	1.0
1	1.45 (1.37 , 1.53)
2	2.02 (1.91 , 2.14)
>2	3.07 (2.88 , 3.27)
**Interaction**	
White#0.cardio-metabolic comorbidity (ref)	1.00
South Asian#1.cardio-metabolic comorbidity	1.12 (0.89, 1.41)
South Asian #2. cardio-metabolic comorbidity	1.01 (0.80, 1.29)
South Asian #3.cardio-metabolic comorbidity	1.18 (0.93, 1.50)
Black#1. cardio-metabolic comorbidity	1.17 (0.85, 1.61)
Black#2. cardio-metabolic comorbidity	1.24 (0.90, 1.71)
Black#3. cardio-metabolic comorbidity	1.24 (0.87, 1.77)
Other #1. cardio-metabolic comorbidity	0.98 (0.80, 1.20)
Other#2. cardio-metabolic comorbidity	1.04 (0.84, 1.29)
Other#3. cardio-metabolic comorbidity	0.95 (0.75, 1.19)
p (interaction)	0.807

*adjusted for age and sex

Norris T, *et al. Heart* 2021;0:1–9. doi: 10.1136/heartjnl-2021-320047

Table S5. Association between individual cardio-metabolic morbidities, in-hospital cardiovascular and renal complications and death

	**Primary outcomes**		**Secondary outcomes**						
	**Any complication**	**Death**	**Heart failure**	**Arrhythmia**	**Cardiac Ischaemia**	**Cardiac arrest**	**Coagulation complications**	**Stroke**	**Renal injury**
	**OR (95% CI)**		**OR (95% CI)**	**OR (95% CI)**	**OR (95% CI)**	**OR (95% CI)**	**OR (95% CI)**	**OR (95% CI)**	**OR (95% CI)**
**Individual baseline cardio-metabolic comorbidities***									
Unadjusted									
Diabetes	1.64 (1.57, 1.71)	1.34 (1.28, 1.40)	1.59 (1.44, 1.75)	1.25 (1.16, 1.35)	1.55 (1.33, 1.81)	1.45 (1.28, 1.64)	1.23 (1.11, 1.37)	1.21 (1.04, 1.40)	1.78 (1.70, 1.87)
Chronic cardiac disease	2.18 (2.10, 2.26)	2.08 (2.00, 2.16)	7.85 (7.12, 8.65)	2.04 (1.92, 2.17)	3.74 (3.25, 4.29)	1.45 (1.30, 1.62)	1.14 (1.04, 1.26)	1.46 (1.29, 1.65)	1.81 (1.73, 1.89)
Hypertension	1.81 (1.75, 1.88)	1.53 (1.48, 1.59)	2.21 (2.03, 2.42)	1.46 (1.37, 1.55)	1.89 (1.65, 2.17)	1.42 (1.27, 1.58)	1.14 (1.05, 1.25)	1.56 (1.38, 1.76)	1.91 (1.83, 1.99)
Chronic kidney disease	2.49 (2.38, 2.60)	2.08 (1.98, 2.17)	3.06 (2.80, 3.34)	1.49 (1.39, 1.61)	2.10 (1.80, 2.44)	1.26 (1.10, 1.45)	1.19 (1.06, 1.33)	1.15 (0.98, 1.35)	3.07 (2.92, 3.22)
Obesity	1.38 (1.32, 1.45)	0.82 (0.77, 0.86)	1.30 (1.16, 1.46)	1.43 (1.32, 1.55)	1.24 (1.03, 1.49)	1.27 (1.10, 1.47)	2.16 (1.95, 2.39)	0.81 (0.67, 0.99)	1.40 (1.32, 1.48)
*Adjusted***									
Diabetes	1.48 (1.42, 1.55)	1.19 (1.13, 1.24)	1.50 (1.36, 1.65)	1.16 (1.08, 1.25)	1.38 (1.18, 1.61)	1.26 (1.11, 1.43)	1.15 (1.03, 1.27)	1.10 (0.95, 1.28)	1.61 (1.53, 1.69)
Chronic cardiac disease	1.69 (1.63, 1.76)	1.21 (1.16, 1.26)	5.77 (5.20, 6.39)	1.74 (1.63, 1.86)	3.03 (2.61, 3.51)	1.25 (1.11, 1.40)	1.27 (1.15, 1.41)	1.11 (0.97, 1.39)	1.39 (1.33, 1.46)
Hypertension	1.41 (1.35, 1.46)	0.97 (0.93, 1.01)	1.54 (1.41, 1.69)	1.22 (1.14, 1.30)	1.43 (1.24, 1.65)	1.16 (1.04, 1.30)	1.17 (1.06, 1.28)	1.22 (1.08, 1.39)	1.50 (1.43, 1.57)
Chronic kidney disease	2.02 (1.93, 2.11)	1.32 (1.26, 1.39)	2.12 (1.93, 2.32)	1.28 (1.19, 1.39)	1.66 (1.42, 1.94)	1.10 (0.95, 1.26)	1.35 (1.20, 1.52)	0.91 (0.77, 1.07)	2.58 (2.45, 2.71)
Obesity	1.83 (1.73, 1.92)	1.34 (1.26, 1.43)	2.13 (1.89, 2.40)	1.69 (1.55, 1.84)	1.62 (1.34, 1.96)	1.44 (1.24, 1.67)	2.03 (1.82, 2.25)	0.99 (0.81, 1.21)	1.81 (1.70, 1.93)

*separate models; ** adjusted for sex, age, and ethnicity

Table S6. Association between baseline cardio-metabolic comorbidities and 'cardiovascular and renal complications or all-cause death'

	**Cardiovascular and renal complications or all-cause death**
	**OR (95% CI)**
*Any cardio-metabolic comorbidity (ref: No)**	1.63 (1.57, 1.70)
*Number cardio-metabolic comorbidities (ref: 0)**	
1	1.34 (1.28, 1.40)
2	1.69 (1.61, 1.77)
>2	2.38 (2.25, 2.51)
*Individual baseline cardio-metabolic comorbidities**	
Diabetes	1.41 (1.36, 1.47)
Chronic cardiac disease	1.51 (1.46, 1.57)
Hypertension	1.21 (1.17, 1.26)
Chronic kidney disease	1.78 (1.70, 1.86)
Obesity	1.65 (1.57, 1.74)

*separate models, adjusted for sex, age, and ethnicity

Table S7. Association between ethnicity and 'cardiovascular and renal complications or all-cause death'

		Cardiovascular and renal complications or all-cause death
		**OR (95% CI)**
Ethnicity (ref: White)		
	South Asian	1.19 (1.11, 1.29)
	Black	1.42 (1.27, 1.59)
	Other	1.04 (0.97, 1.12)

Norris T, *et al. Heart* 2021;0:1–9. doi: 10.1136/heartjnl-2021-320047

Table S8. Associations between baseline cardio-metabolic multi-morbidity, in-hospital cardiovascular and renal complications and death

	Death
	**OR (95% CI)**
**Baseline cardio-metabolic conditions and in-hospital complications**	
0 cardio-metabolic condition, 0 in-hospital complications *(ref)*	1.00
≥ 1 cardio-metabolic conditions, 0 in-hospital complications	1.20 (1.13, 1.27)
0 cardio-metabolic conditions, ≥ 1 in-hospital complications	3.92 (3.57, 4.29)
≥ 1 cardio-metabolic conditions, ≥ 1 in-hospital complications	3.17 (2.98, 3.37)

Adjusted for age, sex and ethnicity

Figure S1. Flow diagram outlining how final sample was derived

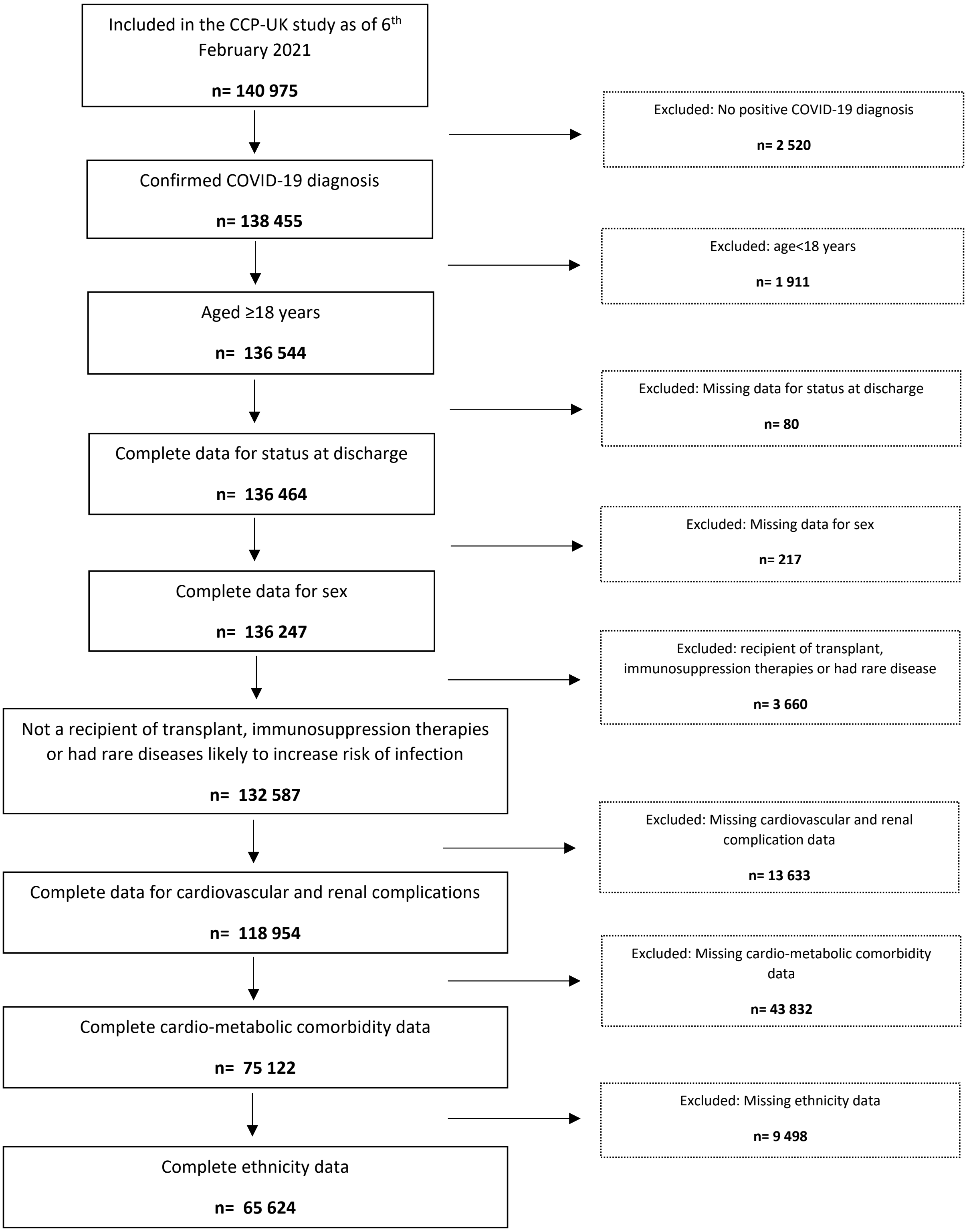

Figure S2. Association between any cardio-metabolic comorbidity and any cardiovascular or renal complication: modification by ethnicity

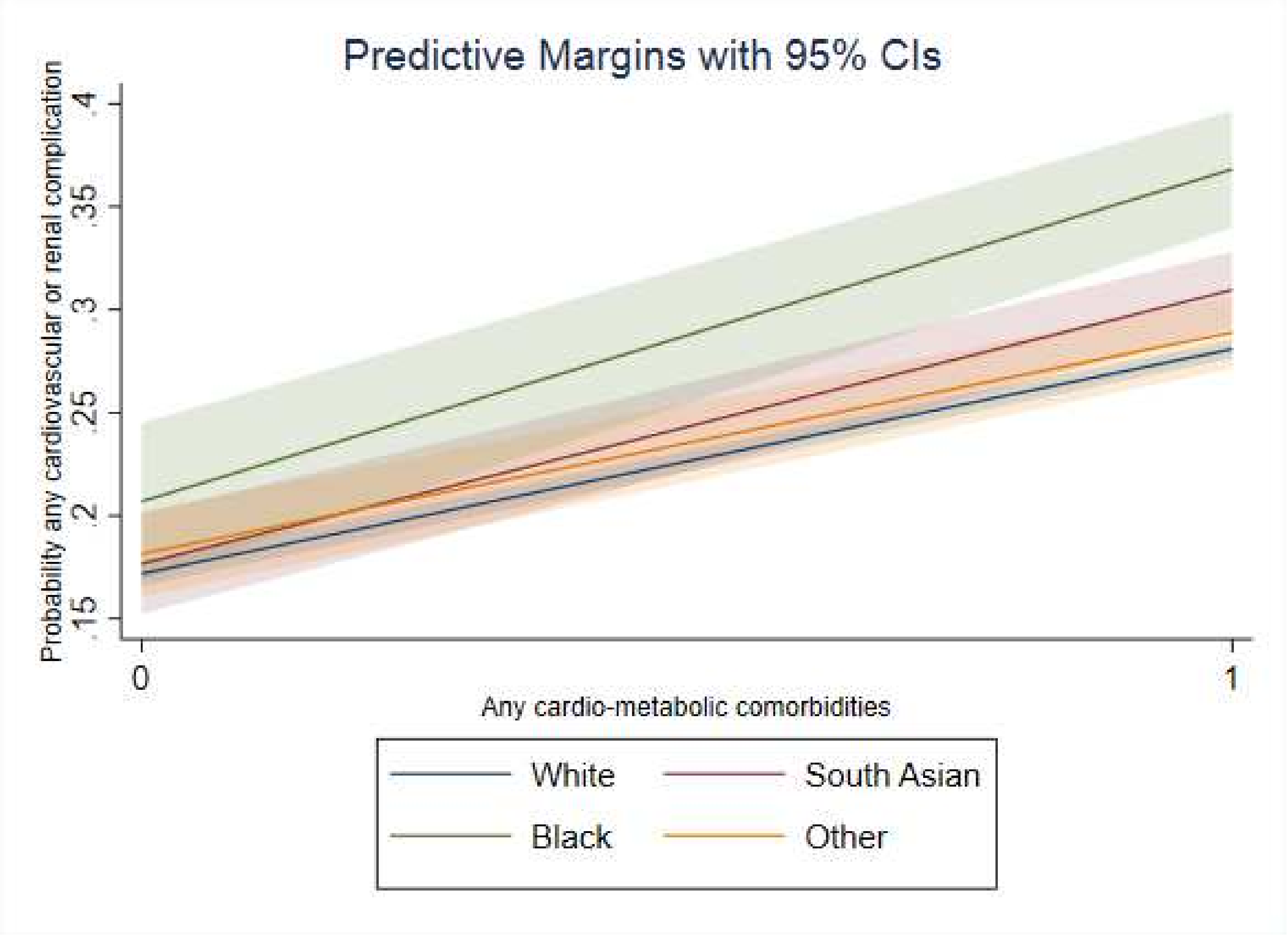

Figure S3. Association between cardio-metabolic multi-morbidity and any cardiovascular or renal complication: modification by ethnicity

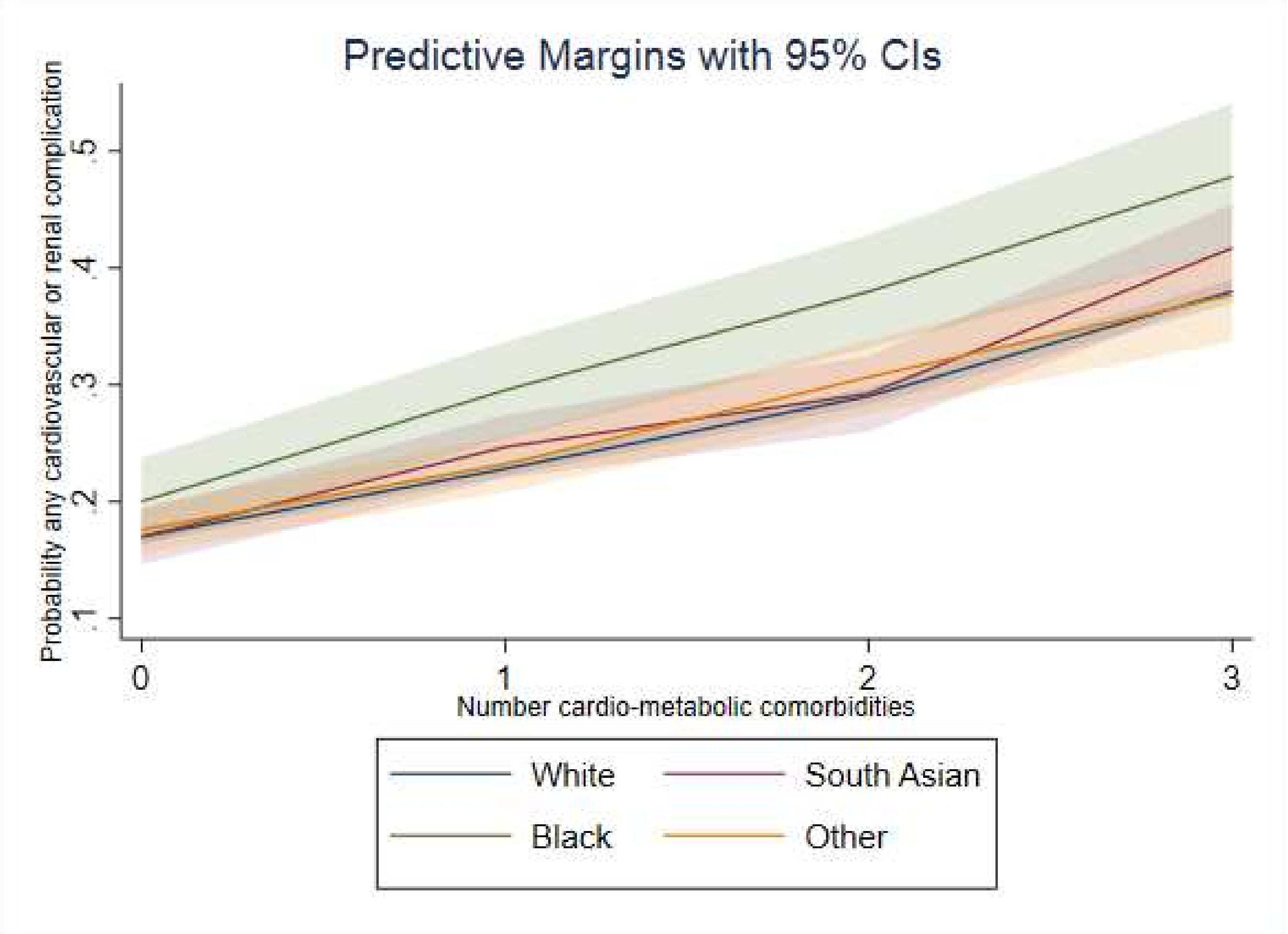

Figure S4. Association between any cardio-metabolic comorbidity and ‘cardiovascular and renal complications or all-cause death’: modification by ethnicity

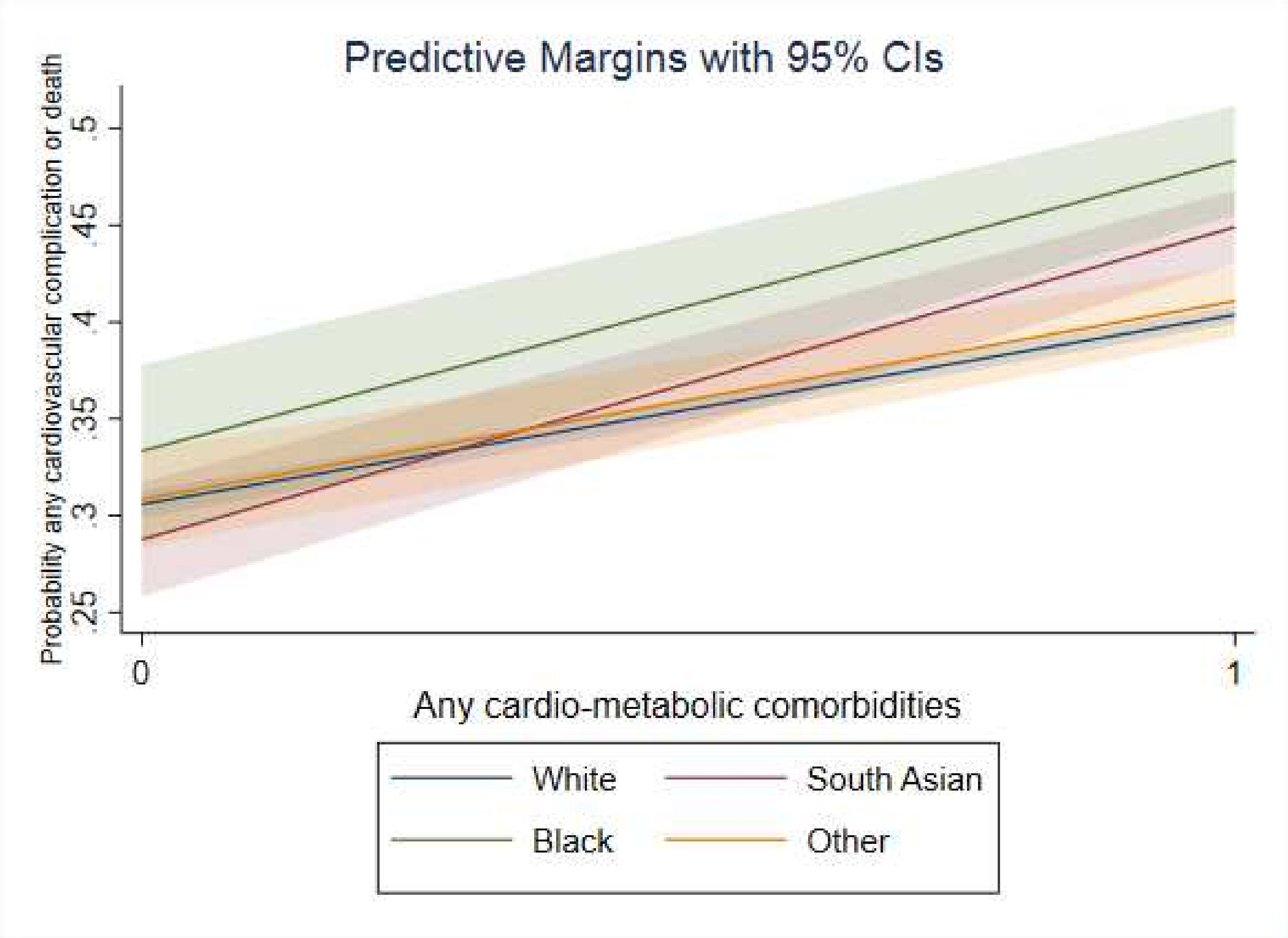

Figure S5. Association between cardio-metabolic multi-morbidity and 'cardiovascular and renal complications or all-cause death': modification by ethnicity

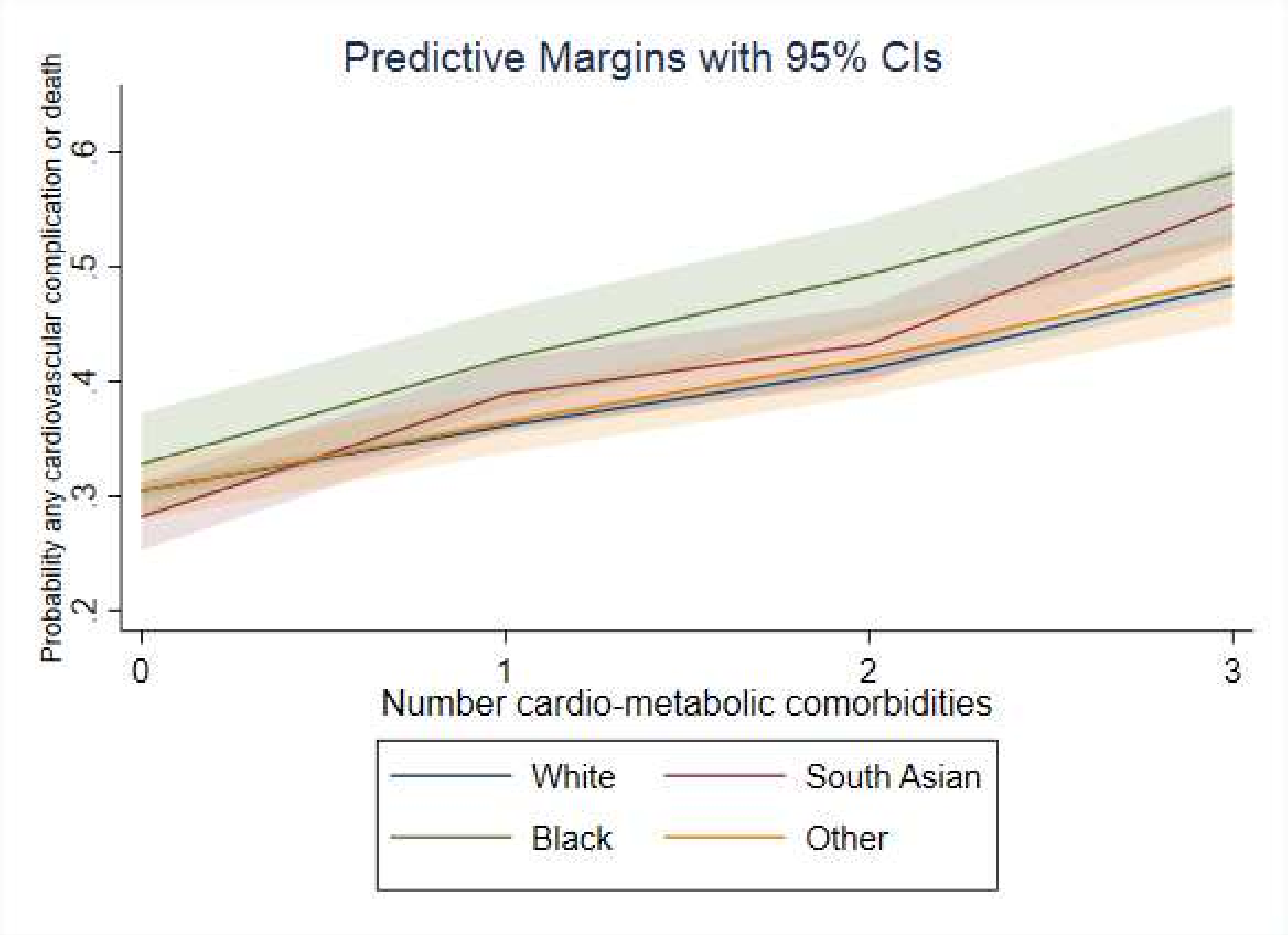

Supplement: Supplementary file 1 [file heartjnl-2021-320047supp001.pdf]
